# Direct Interaction between Carcinoma Cells and Cancer Associated Fibroblasts for the Regulation of Cancer Invasion

**DOI:** 10.3390/cancers7040876

**Published:** 2015-10-14

**Authors:** Hideki Yamaguchi, Ryuichi Sakai

**Affiliations:** 1 Division of Refractory and Advanced Cancer, National Cancer Center Research Institute, 5-1-1 Tsukiji, Chuo-ku, Tokyo 104-0045, Japan; hidyamag@ncc.go.jp; 2 Department of Biochemistry, Kitasato University School of Medicine, 1-15-1 Kitasato, Minami-ku, Sagamihara, Kanagawa 252-0374, Japan

**Keywords:** cancer-associated fibroblasts, cancer invasion, scirrhous gastric carcinoma, peritoneal dissemination

## Abstract

The tumor stroma acts as an essential microenvironment of the cancer cells, which includes many different types of non-cancerous cells and the extracellular matrix (ECM). Stromal fibroblasts (SFs) are the major cellular constituents of the tumor stroma and are often called cancer-associated fibroblasts (CAFs). They are often characterized by α-smooth muscle actin (αSMA) expression, which is indicative of the myofibroblast phenotype and strong contractility. These characteristics contribute to the remodeling and stiffening of the stromal ECM, thereby offering an appropriate field for cancer cell invasion. Importance of the tumor stroma in cancer progression has recently been highlighted. Moreover, several reports suggest that stromal fibroblasts interact with adjacent cancer cells through soluble factors, exosomes, or direct cell-cell adhesion to promote cancer cell invasion. In this review, current models of the regulation of cancer cell invasion by surrounding fibroblasts are summarized, including our recent work on the interaction between stromal fibroblasts and scirrhous gastric carcinoma (SGC) cells by using a three-dimensional (3D) culture system. Further mechanistic insights into the roles of the interaction between cancer cells and stromal fibroblasts in cancer invasion will be required to identify novel molecular targets for preventing cancer cell invasion.

## 1. Introduction

Tumor tissues contain not only cancer cells but also other cellular and non-cellular components. Cellular components of a tumor microenvironment include fibroblasts, immune cells, endothelial cells, mesenchymal stem cells, while non-cellular components include extracellular matrix and deposited growth factors and signaling molecules. These tumor stroma components that surround the cancer cells create the so-called tumor microenvironment that supports the malignant aspects of cancer cells [1,2,3,4,5,6].

Cancer-associated fibroblasts (CAF) comprise a major part of the cellular components of the tumor microenvironment [7,8,9]. CAFs seem to be derived from resident stromal fibroblasts and fibroblast-like cells, such as hepatic and pancreatic stellate cells, and circulating bone marrow-derived cells. Moreover, it is also proposed that epithelial, endothelial, and smooth muscle cells transdifferentiate into CAFs [10,11]. Cancer cells induce the conversion of these various types of cells into CAFs. Through the so-called “education” by cancer cells, CAFs acquire the properties of myofibroblasts including expression of smooth muscle alpha-actin (SMA) and strong contractility [12,13]. CAFs secrete several signaling molecules to stimulate cancer cells and other cell types in tumor microenvironments [14] and remodel the extracellular matrix by secreting ECM components and matrix-degrading enzymes and by physically contracting matrix [12,15]. These properties of CAFs support the malignant progression of tumors by promoting growth, survival, angiogenesis, inflammation, drug resistance, and invasion and metastasis of tumors.

In this review, we would like to focus on the role of CAFs in promoting cancer cell invasion with a particular interest in the direct interaction between CAFs and cancer cells. We also discuss possible strategies to target the interaction between CAFs and cancer cells for the development of new cancer therapeutics.

## 2. CAFs Create Favorable Microenvironments for Carcinoma Cell Invasion

Cancer cell invasion into the surrounding normal tissues is a prerequisite for distant metastasis and is initiated by the detachment of cancer cells from the primary tumor [16,17]. This requires the loss of cell-cell adhesion, breakdown of the basement membrane surrounding the tumor tissues, and cell migration into the tumor stroma [18,19,20]. Phenotypic changes that confer these capabilities on cancer cells are largely induced by epithelial-mesenchymal transition (EMT) [21]. EMT causes the loss of E-cadherin expression, the increase in matrix metalloproteinase production, and the activation of cellular machinery for cell migration.

CAFs secrete soluble factors, including TGF-β and HGF, which promote EMT of the neighboring cancer cells via paracrine signaling [22]. Apart from the soluble factors, exosomes mediate the transfer of functional molecules from CAFs to cancer cells, which stimulate the invasive and metastatic potencies of cancer cells [23,24]. CAFs also secrete matrix-degrading enzymes such as matrix metalloproteinases (MMPs) for ECM degradation. MMPs, which are activated on the invasive front of cancer cells, cleave ECM components within the basement membrane and tumor stroma to generate paths for cancer cell invasion [18]. Cleavage of ECM components also releases latent growth factors in the ECM that in turn promote cancer cell invasion. Invadopodia are actin-rich membrane protrusions formed by invasive cells that focalize MMP activity to the site of ECM degradation [25]. Goicoechea *et al.* recently reported that an actin-binding protein palladin promotes invasion of cancer cells by enhancing invadopodia formation in CAFs [26].

Matrix stiffening in the tumor microenvironment enhances cancer cell migration and invasion through integrin-mediated mechanotransduction [27,28,29]. CAFs also contribute to matrix stiffening by secreting ECM components and by directly and mechanically contracting ECM through actomyosin contractility [15]. Recent studies showed that the functions of Cav1 and YAP in CAFs are required for matrix stiffening, which in turn induces cancer cell invasion [30,31].

## 3. Direct Interaction between CAFs and Carcinoma Cells Controls Invasion

**Figure 1:**
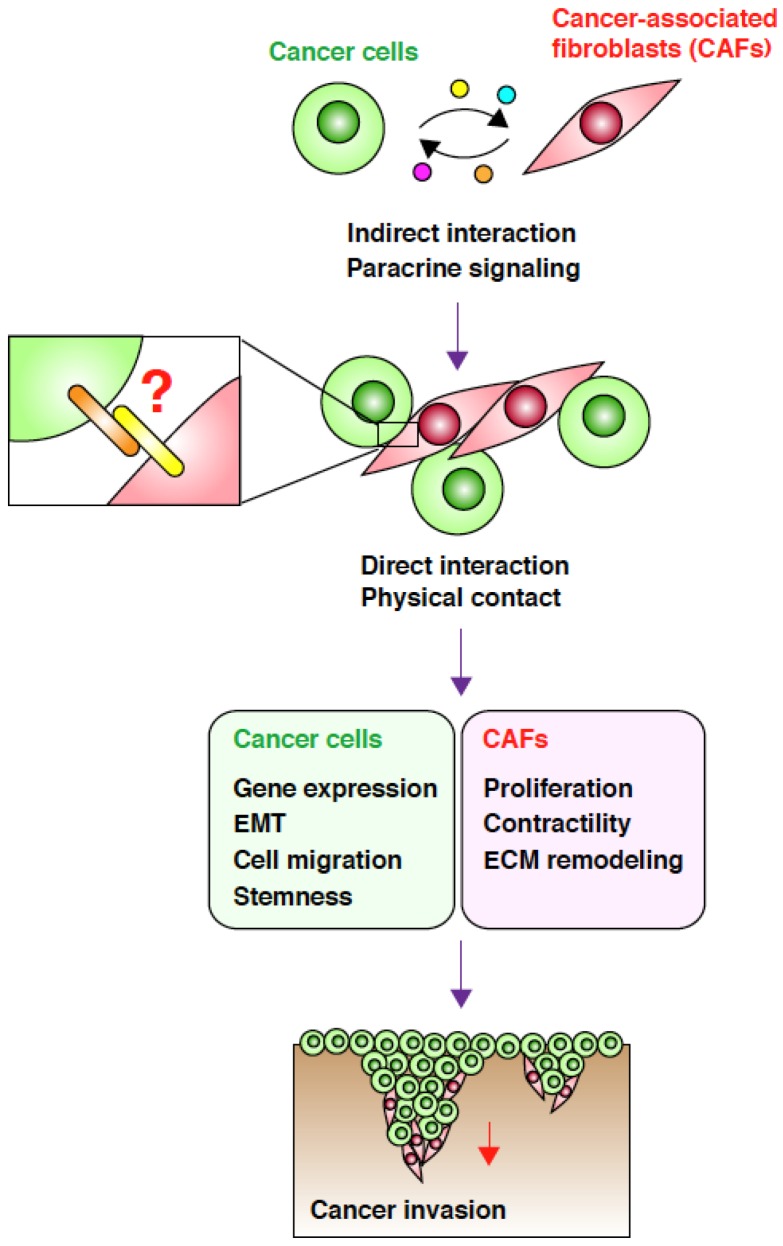
Direct interaction between cancer-associated fibroblasts (CAFs) and cancer cells promotes cancer cell invasion. CAFs and SGC cells indirectly interact via paracrine signaling mediated by soluble factors and exosomes. This interaction induces phenotypic changes in both the cell types, which in turn trigger cancer cell invasion. Through paracrine signaling, the two cell types attract each other, leading to a direct physical interaction that may be mediated by cell-surface adhesion molecules. This direct interaction may cause further changes in both cell types, resulting in a more efficient CAF-led cancer cell invasion.

In addition to paracrine communication via soluble factors and exosomes, accumulating evidence highlights the importance of direct physical interactions between CAFs and cancer cells for enhancement of cancer cell invasion (Figure 1). An important study by Gaggioli *et al.* showed that CAFs lead the invasion of squamous cell carcinoma (SCC) cells by generating tracks in the ECM matrix in a three-dimensional (3D) co-culture system [32]. Direct observation of the invading cells revealed that the leading cells are always CAFs and that SCC cells associate with and follow CAFs to co-invade as collective chains. Importantly, a conditioned medium of CAFs was not able to enhance cancer cell invasion. Furthermore, separation of the two cell types with a thin matrix markedly blocked co-invasion. These observations suggest that close proximity, and probably direct contact, between CAFs and SCC cells is required for SCC cell invasion. More recently, Otomo *et al.* demonstrated that p53-depleted CAFs enhanced invasion of lung carcinoma cells in a 3D co-culture system and that this process also requires direct contact between the two cell types [33].

Scirrhous gastric carcinoma (SGC), a subtype of diffuse-type gastric adenocarcinoma, has a very poor prognosis [34,35]. SGC is characterized by rapid and diffusive invasion under the submucosa, strong fibrosis associated with massive growth of fibroblasts, and frequent peritoneal dissemination. CAFs have been shown to promote the aggressive phenotypes of SGC cells [36], which includes growth and tumorigenicity [37], migration and invasion [38,39], adhesion to mesothelial cells [40], peritoneal dissemination [41,42], and stemness [43]. Conversely, SGC cells stimulate the growth of CAFs [44] and induce contraction of CAFs, leading to matrix stiffening [45]. These reports attribute the communication between CAFs and SGC cells to paracrine signaling. Nevertheless, Semba *et al.* showed that the direct interaction between stromal fibroblasts and SGC cells is required for induction of fibroblast proliferation and for the development of invasive phenotypes in SGC cells [38].

**Figure 2:**
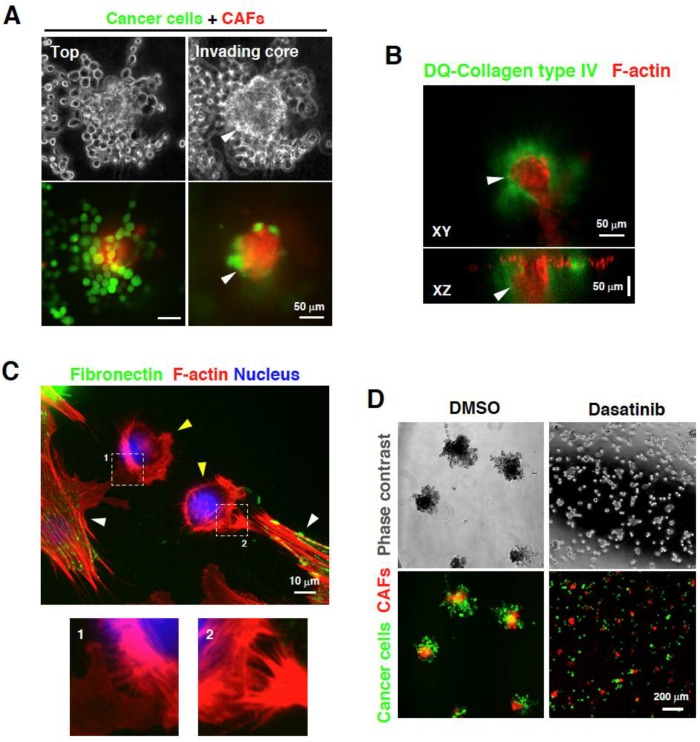
Direct interaction between cancer-associated fibroblasts (CAFs) and scirrhous gastric carcinoma (SGC) cells. (**A**) CAFs and SGC cells cultured at the top of the 3D Matrigel form invasive foci. SGC cells come in contact with CAFs and co-invade the 3D Matrigel; (**B**) F-actin staining of the invasive foci consisting of CAFs and SGC cells showed that the foci invade the Matrigel and are associated with cleaved signals for collagen type IV; (**C**) CAFs expressing fibronectin (white arrowheads) physically associate with SGC cells by extending their lamellipodia and filopodia toward SGC cells (yellow arrowheads). The lower panels show magnified images of the boxed regions; (**D**) Treatment with an Src inhibitor dasatinib blocks the interaction between CAFs and SGC cells, resulting in the suppression of invasive foci formation and invasion. (**A** and **D**) Images are reproduced from Yamaguchi *et al.* [46].

We recently reported that co-culturing SGC cells and CAFs derived from SGC tissues on 3D Matrigel induce formation of foci that contain the two cell types in close contact and invade the Matrigel [46] (Figure 2A,B). CAFs localized at the center and the leading front of the invasive foci and the associated SGC cells co-invaded the underlying matrix. Interestingly, SGC cells alone did not show a strong invasive phenotype. Satoyoshi *et al.* recently reported similar observations that CAFs lead and co-invade with SGC cells in a 3D co-culture system and *in vivo* [47]. This phenomenon was not recapitulated by the addition of a conditioned medium of CAFs to SGC cells, or *vice versa*, indicating the importance of a direct interaction between the two cell types. Indeed, time-lapse imaging of the invasive process revealed that both cells attract each other and extend long protrusions through which they subsequently associate. Additionally, direct interaction between the two cell types through filopodia and lamellipodia-like structures was observed in 2D co-culture (Figure 2C).

We also showed that direct interaction with SGC cells promotes the ability of CAFs to remodel and mechanically disrupt the ECM matrix through an increase in actomyosin contractility. These observations show that SGC cells directly interact with CAFs and promote their ability to mechanically remodel the ECM matrix for invasion. As strong fibrosis and contraction of the stomach wall occur during SGC progression, the matrix remodeling activity of CAFs stimulated by SGC cells may contribute to the pathological features of SGC.

The molecular mechanisms by which the direct physical interaction between CAFs and cancer cells stimulates cancer cell invasion remain poorly understood. It was reported that CAFs enhance EMT and cell migration of non-small cell lung cancer (LSCLC) cells more potently through direct contacts rather than through indirect interactions [48]. Gene expression of breast cancer cells that are in direct contact with CAFs markedly differ from that observed in cells that indirectly interact with CAFs through soluble factors [49]. Therefore, direct contact between the two cell types may elicit specific changes in gene expression that induce EMT and confer the migratory phenotype on the cancer cells.

Cancer cells that have undergone EMT acquire stem cell-like traits and have a propensity to invade [21]. Kinugasa *et al.* reported that CAFs sustain the stemness of colorectal cancer stem cells, which requires direct cell-cell contact between CAFs and cancer stem cells and CD44 expression in CAFs [50]. It is unclear whether CD44 is involved in the direct interaction or whether it just participates in maintaining stemness. Although this study did not examine the invasiveness of cancer cells, the induction of stemness by CAFs through direct interactions may be associated with increased invasive potencies.

An interesting study showed that a conditioned medium of co-cultured stromal fibroblasts and breast cancer cells increases migration, invasion, and metastasis of cancer cells, whereas that of homotypic cultures had little effect on these cancer progression-associated properties [51]. Surprisingly, this effect of co-cultured conditioned medium can be induced by transient treatment of cancer cells and is mediated by a TGF-β mediated mechanism. This observation suggests that the direct interaction also affects paracrine signaling between CAFs and cancer cells.

## 4. Targeting the Interaction between CAFs and Carcinoma Cells

Several studies corroborated the therapeutic potential of targeting tumor progression-supporting functions of CAFs [52,53]. For example, targeting CAFs themselves by eliminating FAP-positive CAFs in mouse models suppressed tumor growth in lung and pancreatic carcinomas [54]. Several agents that have been tested in preclinical studies or clinical trials target the soluble mediators of the interactions between CAFs and cancer cells, including HGF, TGF-β, and CXCL12 [52]. In the case of SGC, COX2 inhibitor was shown to block CAF-stimulated SGC invasion and metastasis [55]. Pro-invasive functions of CAFs may also be targeted to block CAF-driven carcinoma cell invasion. Chemical screening of CAF contraction inhibitors identified lovastatin and simvastatin as inhibitors of CAF-stimulated SCC invasion [56].

To identify inhibitors of the direct interaction between CAFs and cancer cells, we utilized the above-mentioned 3D co-culture system of CAFs and SGC cells for drug screening [46]. Thus, we found that an Src inhibitor dasatinib effectively blocks the physical association between CAFs and SGC cells with minimal cytotoxic effect (Figure 2D). Dasatinib showed marked therapeutic potencies against peritoneal dissemination of SGC in mouse model experiments. Importantly, histological analysis revealed that metastasized tumors are less associated with stromal fibroblasts in mice treated with dasatinib as compared to that observed in control mice. These results demonstrate that the direct interaction between CAFs and SGC cells can be a target for anti-metastasis therapy.

When targeting CAFs, it should be considered that they may also play suppressive roles in tumor progression. Two recent studies demonstrated that depletion of CAFs in mouse models accelerates progression of pancreatic cancer [57,58]. These results contradict other studies [54,59,60] and this discrepancy could be caused by the use of different animal models (e.g., genetically-engineered, human-to-mouse xenograft) or different approaches to deplete CAFs (e.g., depletion of FAP or αSMA positive cells, sonic hedgehog inhibition). Given the heterogeneous nature of CAFs [9], it is also possible that their subpopulations have tumor-suppressive functions. Alternatively, their roles may differ between tumor types, stages, and genetic backgrounds. Further studies are needed to understand intra- and inter-tumoral heterogeneity of CAFs for the development of strategy to appropriately target tumor-promoting functions of CAFs.

## 5. Conclusions and Perspectives

Over the last few decades, critical roles of CAFs in the promotion of cancer invasion have been well established. Extensive efforts have been made to understand the molecular mechanisms underlying the interaction between CAFs and cancer cells during cancer cell invasion. However, the importance of the direct interaction between the two cell types has emerged only in recent years; therefore, several questions remain to be answered. Probably, the most important challenge is to identify molecules that mediate the physical association of CAFs with cancer cells. As these molecules are supposed to be displayed on the cell surface, they are good targets for the development of new molecular targeted therapies. It is also important to identify signaling pathways and molecules in cancer cells that can be activated upon direct interaction with CAFs. Further analysis of gene expression profiles and activation statuses of signaling molecules and characterization of cellular structures mediating cell-cell adhesion may help identify molecules involved in cancer invasion promoted by the direct interaction between CAFs and cancer cells.
